# Anomaly Detection and Segmentation in Measurement Signals on Edge Devices Using Artificial Neural Networks

**DOI:** 10.3390/s25175526

**Published:** 2025-09-05

**Authors:** Jerzy Dembski, Bogdan Wiszniewski, Agata Kołakowska

**Affiliations:** Faculty of Electronics, Telecommunications and Informatics, Gdańsk University of Technology, Narutowicza 11/12, 80-233 Gdańsk, Poland; jerzy.dembski@pg.edu.pl (J.D.); bogdan.wiszniewski@pg.edu.pl (B.W.)

**Keywords:** time-series data cleaning, intelligent IoT sensors, constrained end devices

## Abstract

In this paper, three alternative solutions to the problem of detecting and cleaning anomalies in soil signal time series, involving the use of artificial neural networks deployed on in situ data measurement end devices, are proposed and investigated. These models are designed to perform calculations on MCUs, characterized by significantly limited computing capabilities and a limited supply of electrical power. Training of neural network models is carried out based on data from multiple sensors in the supporting computing cloud instance, while detection and removal of anomalies with a trained model takes place on the constrained end devices. With such a distribution of work, it is necessary to achieve a sound compromise between prediction accuracy and the computational complexity of the detection process. In this study, neural-primed heuristic (NPH), autoencoder-based (AEB), and U-Net-based (UNB) approaches were tested, which were found to vary regarding both prediction accuracy and computational complexity. Labeled data were used to train the models, transforming the detection task into an anomaly segmentation task. The obtained results reveal that the UNB approach presents certain advantages; however, it requires a significant volume of training data and has a relatively high time complexity which, in turn, translates into increased power consumption by the end device. For this reason, the other two approaches—NPH and AEB—may be worth considering as reasonable alternatives when developing in situ data cleaning solutions for IoT measurement systems.

## 1. Introduction

Monitoring of environmental conditions, such as the risk of forest fires, the condition of flood embankments along rivers, and soil parameters in precision agriculture, requires the collection of data from vast and often hard to access areas; for this purpose, in situ measurement sensors can be utilized. Such areas usually lack an efficient telecommunications infrastructure enabling the continuous transmission of the acquired measurement data for the purpose of delivering them to a processing center (e.g., a cloud computing center). Due to the excessive costs of establishing such infrastructure, the collection of data from sensors scattered throughout an extended area currently requires the use of an unmanned aerial vehicle (UAV), acting as a mobile go-between serving both sensors and the cloud, which is piloted from the ground or operates autonomously. In such a nomadic scheme, a gateway onboard the UAV receives chunks of data from the sensors it flies over, which are then stored in its local memory and, after landing, uploaded to the cloud via the nearest available access point.

The development of such a measurement ecosystem poses a number of serious challenges. First, each sensor on the ground has a limited time window to send measurement data to the UAV. Wireless communication protocols used in IoT use relatively small packet sizes to conserve energy and maximize battery life. Moreover, as more devices can transmit data independently within the same time frame, congestion/collisions are reduced by decreasing data rate managed by the protocol’s underlying airtime allowance mechanism [1]. As a result, the transmission of large amounts of data from a sensor may not fit within the available time window of a passing UAV. To reduce the volume of data to be sent, they must be cleaned of erroneous, misleading, and otherwise redundant data, all of which must be performed by a constrained end device with limited computing, memory, and power resources. Measurement data of soil signals are stored in the sensor’s memory as time series of values reflecting consecutive samples. The literature lacks generic solution patterns which can be readily used for the automatic detection and localization (i.e., segmentation) of sample fragments in signals, which is necessary to implement the above-mentioned operations. The main reason for this shortcoming is that sufficiently large training datasets are required to effectively train ML models for such tasks. For instance, in [2] a combined LSTM–Autoencoder was proposed, which can detect anomalies in indoor air quality data by learning long-term dependencies and reconstructing sequences to identify deviations. In turn, solutions using 1D U-Net models described in the literature (e.g., [3]) are especially effective for training on small datasets thanks to their symmetric and compact design. However, when compared with autoencoders, their memory footprint is higher due to deeper layers and skip connections, are more energy-intensive due to more layers and operations, and require more capable CPUs. Thus, although more powerful for segmentation tasks, they are better suited for more capable cloud instances.

The inspiration for this study is the belief that, when there is insufficient data for automatic analysis using state-of-the-art deep learning methods, plain visual interpretation of signal plots to determine any common-sense abnormal change-points and patterns may be misleading [4]. Intuition tells us that, similarly to the synoptic interpretation of weather maps, only in-depth knowledge of the ongoing processes whose parameters we measure will allow us to correctly interpret their recorded values. One example could be the problem of detecting power gaps (as described later in Table 1), resulting in misplaced samples. Based on this knowledge, augmentation of real data may be performed by mimicking the relevant physical processes in the sensor’s operational environment. A key novel component in such automation that is that explainable anomalies can be represented, in a meaningful way, using instances from just a handful of their parameterized generic classes. For sets of realistic data generated in such a manner, four alternative models for detecting and segmenting fragments of anomalous samples in soil signal time series, with different dynamics and physics, were evaluated, from unoptimized and optimized heuristic detectors, through to combined neural–heuristic ones and up to two variants of autoencoders. Each of these models can be implemented on an Arduino-based constrained device.

The results of our study are significant for the IoT research community and practitioners for two reasons. The first is that we can effectively cope with the problem of insufficient volumes of training data by augmenting them based on the comprehensive physics-informed ML (PIML) approach [5]. The anomalies present in time-series of measurement data generated in this way are not merely injected perturbations, and can provide a sound basis for ensuring the ecological validity of the tested models’ performance under unpredictable, real-world conditions. The second is showing how cheap smart sensors can significantly reduce the costs of implementing measurement ecosystems over large areas without the need to invest into associated telecommunications infrastructure. Our field tests in real farmland and forest environments show that with an appropriate wireless telecommunication technology (e.g., LoRaWAN), constrained battery-powered IoT end devices can maintain operational readiness for long periods due to only the sporadic transmission of minimal byte loads for short distances between the ground and the UAVs flying by being required.

The remainder of this paper is structured as follows: In Section 2, we set the scene for the rest of the paper from the perspective of a nomadic in situ soil data acquisition scheme using UAVs and constrained end devices, which we implemented as a Rural IoT system. In Section 3, three basic neural network architecture models and their implementations investigated in the study are described. In Section 4, the results of our experimental evaluation of their suitability and effectiveness in detecting various classes of anomalies in the time series comprising measurement signals of several key soil parameters are presented and discussed. Finally, in Section 5, our results regarding the use of neural network models for the intelligent segmentation of time-series data are summarized and directions for future work are detailed, with the goal of increasing the technological readiness level of our soil measurement sensors in their final (production) version.

## 2. Detection and Segmentation Problem

The traditional method for detecting anomalies in measurement signals involves detecting atypical signal fragments in relation to the typical waveform. Furthermore, the segmentation problem involves precisely locating the signal fragment where the anomaly occurs; it can therefore be said that segmentation is a more general and more difficult problem than detection itself.

There is a wide spectrum of causes for this type of phenomenon, which have been analyzed in the literature for years. These include natural factors such as varying weather conditions, radiation, lightning, or chemical activity of the monitored environment, as well as human-caused disruptions, which can be unintentional (by other devices or machinery) or intentional (e.g., jamming, spoofing). Examples include the following:

- Environmental disturbances such as sudden weather changes, large fluctuations in pressure, temperature, radiation, fires, or pollution temporarily disrupting normal readings; such disturbances are the major source of anomalies [6,7].
- Hardware-related issues, including sensor aging and battery depletion [8,9] or physical damage to nodes. Moreover, the sensing capabilities of the sensors used in common low-cost configurations are limited, making the measurement values more sensitive to external factors [6,8].
- Communication errors [6,7,8,10] such as packet loss, data corruption, or network congestion compromising data integrity, particularly in a multi-sensor distributed setting. These errors may be caused by interference from other wireless-based technologies, electromagnetic noise, narrow bandwidth degrading communication speed, or physical barriers such as high buildings.
- Software failures stemming from firmware bugs, misconfigurations, or calibration drift over time affecting the accuracy of sensor output. Furthermore, human factors such as incorrect sensor deployment, inadequate measurement techniques, or other operational misuse may contribute to such failures [8,10]; e.g., the interpretation of data might be wrong if seasonal or environmental factors are not considered during the calibration process [10].
- Various types of cyberattacks, including jamming attacks causing communication delays or packet loss [11], as well as spoofing (i.e., the injection of false data) [12].

In rural IoT measurement ecosystems such as those considered in this study, not all of the abovementioned causes of anomalies in the time-series data collected by the nomadic UAV gateway will require equal attention. Certain environmental disturbances or hardware issues, including sensor aging and battery depletion, are undoubtedly more difficult to control than the others, for which we can either deploy appropriate technologies or design sensor usage scenarios which make them practically non-existent. This is particularly true for communication errors when using a properly configured technology that is specifically designed to wirelessly connect battery-powered IoT devices to the Internet (e.g., LoRaWAN) [13]. On the other hand, software errors due to calibration drift over time and measurement inaccuracy practically did not occur during our experiments using sensors with an assumed lifetime spanning over one vegetation period; typically from March to October in European latitudes. Therefore, our sensors were designed as single-use, disposable devices. Finally, when monitoring soil parameters for agricultural purposes, especially with multiple sensors spread over very large areas of arable land, the likelihood of human intrusion is negligible. In this scenario, the UAV itself seems to be at greater risk, especially when its navigation unit uses GNSS signals that can be jammed or spoofed in the event of an attack.

To find unusual patterns in time-series data (e.g., signal samples) that deviate significantly from the typical signal waveforms, it is crucial to understand their underlying physical processes—especially when no analytical model of a given process exists or the amount of measurement data is too small to identify it. To cope with this issue, we utilize the physics-informed ML (PIML) approach [5], which comprised a comprehensive interpretation of the recorded soil signal samples and the development of explainable anomaly classes. Moreover, understanding the phenomena occurring in the soil allowed us to parameterize these anomaly classes as generic classes, allowing us to generate extensive synthetic datasets that included anomalies found in real measurement signals, not just random perturbations injected arbitrarily.

### 2.1. Generic Anomaly Classes

We started by analyzing four soil signals, namely, temperature (T), moisture (M), acidity (pH), and solar radiation (PV), in a volume of over 175,000 samples collected using 7 autonomous measurement sensors at different locations scattered within an area of approximately 35 km during one full growing season (i.e., from April to October) [14]. We have previously detailed the characteristics of their underlying physical processes in [15,16]. For sake of brevity, we reiterate only their essential features below:

T exhibits a strong (regular) daily trend, as solar heat is gradually accumulated in the soil from sunrise and radiated out after sunset;
M has periodicity that is hard to capture, as rainfalls combined with soil/terrain conditions and the location of the sensor may result in flooding of the measurement probe;
pH normally has no trend, as minerals contained in the soil can dissolve only in the water contained therein up to a certain concentration limit (saturated solution);
PV has regular (every 24 h) leading edges of the voltage signal corresponding to two consecutive sunrises, and  rapid fluctuations in between these time points depending on the actual external load (open circuit voltage).

Based on the above understanding, we carried out comprehensive visual interpretation of each signal plot to find and explain signal fragments that deviated from the underlying physical processes. Samples collected from all sensors were stored in the InfluxDB time-series database, from which subsequent portions of data were visualized using a visualization package, Grafana, integrated in our cloud-based CLaaS technology stack [16]. The identified deviations are explained in Table 1.

**Figure 1:**
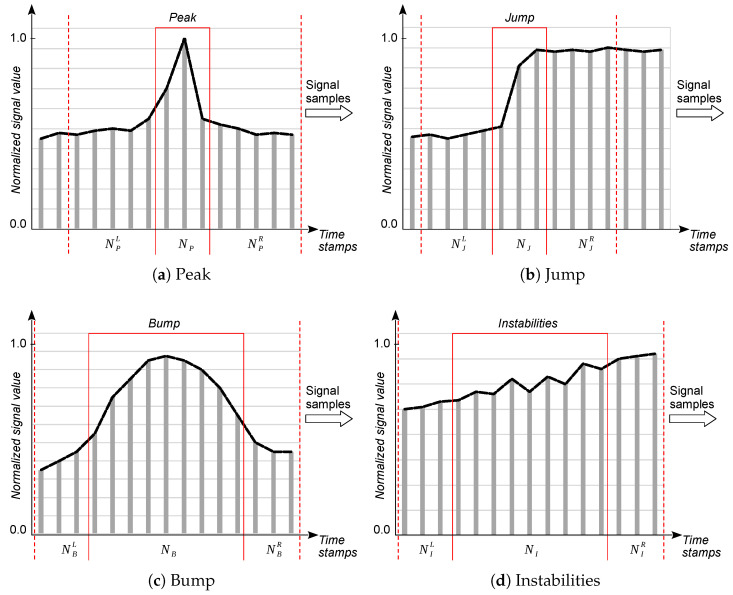
Classes of anomalies in soil signals.

By understanding the physical basis of individual types of anomalies, it is possible to search for methods (both heuristic and neural) for their detection, thus answering whether a given fragment of signal samples contains a sequence of samples that can be classified into one of the types identified in Table 1. Subsequently, we can look for methods for their segmentation, which allow for determination of their most probable positions in the analyzed sequence. Of course, a straightforward solution would be to use an autoencoder that is trained to process the input signal into the same output signal, where the objective function is to minimize the output signal reconstruction error. When processing an unknown signal in this way, an excessive reconstruction error would indicate an anomaly that is either known (explainable) or unknown beforehand. However, a disadvantage of this approach when dealing with a small volume of training data is that a valid signal that was not in the training set may also be considered an anomaly. A false positive error, i.e., detecting an anomaly where there is none, may result in the removal of an important fragment of the signal during its cleaning. Therefore, a more reasonable approach in our case was to study the similarity of a signal fragment to previously established patterns. In this line, we parameterized four types of pattern explained in Table 1; namely, *peaks*, *jumps*, *bumps*, and *instabilities* [15]. These patterns are outlined schematically in Figure 1.

In this paper, we denote each potentially anomalous signal fragment by $N_{*}$, ∗∈{P,J,B,I}, which is referred to as the anomaly center, with left and right neighbors $N_{*}^{L}$ and $N_{*}^{R}$, respectively. Samples delimiting the anomaly neighbors from the associated center are termed the left and right edges of the anomaly, respectively. This parameterization is necessary, as simply detecting the existence of an anomaly is not sufficient in situations where anomalous signal fragments must be cleaned (i.e., either removed or corrected) [15]. In such cases, segmentation of the anomaly requires determining its boundaries.

Detection, segmentation, and cleaning of anomalies in time series of measured signals should be performed locally in the sensor’s processing environment, due to the previously mentioned need to minimize the byte payload transmitted from the sensor to the UAV in the time- and energy-constrained data transmission window [13]. Hence, in this paper, we analyze alternative models for the detection and segmentation of the four anomaly classes specified in Figure 1, which must be deployed on constrained end devices. It is worth noting that sensors in rural IoT ecosystems should be able to deal with anomalies that cannot be cleaned locally, as well as anomalies that can be cleaned with simple filtration; the former are power gaps, whereas the latter are absolute errors.

In the case of power gaps, as explained in Table 1, each sensor can only detect the fact that some samples have been time-shifted. It can do this by counting samples of the T, M, and pH signals between two consecutive PV instant voltage drop-ups, corresponding to two sunrises. With a known sampling period, the number of signal samples should fall within some fixed interval. When the calculated actual number of samples is much lower or higher, it means that the sensor was down for some period. Then, all samples in that time frame should be marked as misplaced and, when uploaded to the cloud instance, can be correctly shifted on the timeline by combining samples from other sensors. One possible way to fix this anomaly is a heuristic time-series data fusion method we have previously developed, which combines the Dynamic Time Warping (DTW) and Derivative DTW (DDTW) methods [17].

In turn, eliminating absolute errors from measurement data requires setting the appropriate limit values for the variability ranges of individual signals in the sensor’s software. For European latitudes, these ranges can be set as pH ∈{3.0,9.0}, M ∈{10%,80%}, and  T ∈{0 °C,40 °C}. When implementing a rural IoT ecosystem at other geographical latitudes, it is sufficient to shift these ranges according to the normative documents of the relevant agricultural authorities (ministries). Moreover, depending on the manufacturer of the cell used, its catalog provides the nominal value of its output voltage (e.g., PV ∈{0.0 V,6.6 V}). When reading the values of subsequent samples that fall outside the allowed ranges, the samples are saved as *empty*. Their values can then be locally calculated as an average of the left and right sample values from the correct range, determined from the signal trend, or simply forwarded to the cloud instance where it can be repaired through fusion with data from other sensors. The sensors in our Rural IoT ecosystem implement the first of these solutions.

### 2.2. Training and Testing Datasets

Measurements carried out in a real operational environment, characterized by highly variable weather conditions for approximately 215 days spanning the three seasons from spring to autumn, provided us with real and representative soil data. However, developing an effective mechanism for automatic detection and segmentation posed a fundamental “chicken and egg” dilemma: should we first collect a significant amount of data and then discover patterns, or determine the meaningful patterns first and then look for them in the analyzed data? The main problem was the high cost of deploying a sufficient number of sensors to collect the initial data, as too small a dataset may not enable the proper training of neural models for anomaly detection and segmentation. Therefore, it was necessary to develop a workaround through the generation of synthetic but realistic data, taking advantage of the explainable physics of phenomena occurring in the soil.

With the generic anomaly models described earlier and, according to the previously discussed daily periodicity of the pH, M, and T signals, we generated sufficiently large training and testing datasets. First, for the original time-series data (raw signals) recorded by each of the seven sensors over the entire growing season, we created seven corresponding reference (ideal) signals by manually removing all pre-determined anomalies. Then, for each reference signal, we generated synthetic signals by injecting different fragments of anomalous samples into the ideal signal time-series data; these were not simple random perturbations injected into reference signals, but entire fragments corresponding to particular types of anomalies. The individual parameters of the anomalies specified in Figure 1 changed within the ranges implied by the physical attributes of the measurement process, and the logic of the reference signals of individual soil parameters, according to an algorithm specifically developed for this purpose (the details of which were presented earlier in [15]). The aforementioned approach has two advantages. First, injecting faults into a dataset gave us an accurate ground truth that helped to better understand the performance of anomaly detection and segmentation methods. Second, we were able to control intensity of each anomaly and, thereby, could explore the limits of performance of each such method and comparatively assess different schemes at low anomaly intensities [9].

The data obtained from three sensors $S_{tr}=\left\{s01,s02,s23\right\}$ and processed as described above were used for training, while data from the remaining four $S_{te}=\left\{s03,s10,s21,s22\right\}$ were used for testing. Within both groups $S_{tr}$ and $S_{te}$, sensors were distributed in different locations to ensure that the data obtained were as representative as possible, ensuring the good generalization ability of the resulting models. All data represented time series for three measured physical soil parameters: pH, M, and T.

### 2.3. Segmentation Quality Assessment

To compare the segmentation algorithm’s output with respect to the known ground truth, we used the intersection over union (IOU) metric given by Formula (1):

(1) $$IOU=\frac{\left|t\cap y\right|}{\left|t\cup y\right|}=\frac{TP}{P+FP}\;,$$

where *t* denotes a sequence of bits containing true anomaly labels, *y* denotes an analogous sequence output by the segmentation algorithm, t∩y is the intersection over individual bits, t∪y is the union over individual bits, and |.| is a sum of bits. In equivalent notation, it is calculated as the number of true positive samples (TP) divided by the sum of all ground truth samples (P) and false positive samples (FP). All results presented in Section 4.2 were calculated for test signals concatenated into one long vector, which allowed us to avoid averaging as, in the case of unevenly distributed anomalies in different signals, it can lead to unreliable results.

Another popular metric used in this context is the F1 metric, which can be represented as an IOU-dependent value obtained from Formula (2):

(2) $$F1=\frac{2IOU}{1+IOU}.$$

If the output of the segmentation system is the probabilities of anomaly classes, it is necessary to adopt a probability threshold above which one may consider that a sample belongs to the anomaly class. Depending on the adopted threshold, different TP and FP values will be obtained, as well as the true positive rate TPR=TP/P, where *P* is the number of positive samples, and the false positive rate FPR=FP/N, where *N* is the number of negative samples. In this case, a reliable approach for evaluation of the segmentation system is using the receiver operating characteristic (ROC) parametric curve, the parameter of which is a threshold value and the related area under curve (AUC) measure can be obtained, which is independent of the selected threshold.

### 2.4. Edge–Cloud Work Distribution

An important issue to be addressed when developing a measurement IoT system is the distribution of work between end devices and their supporting cloud. While the end device (sensor) could detect anomalies in data and send them to the cloud for processing, this would increase its energy consumption due to excess computations. Cloud feedback could optimize such a process by combining data from multiple end devices in a collaborative learning scheme; however, this would require additional bandwidth and energy reserve for the latter to receive local model updates. Balancing this scheme, in fact, requires prior experimentation for each specific application.

Due to the limited computing resources of the end device (e.g., a measurement sensor), development of its software requires support from an external entity, such as a computing cloud. This is particularly important when ML models used by anomaly detectors require the ability to continuously evolve, enabling increasingly better cleaning of measurement data time series as knowledge about the monitored phenomena is gradually accumulated across the entire measurement ecosystem. This knowledge should be periodically redistributed, starting from the initial configuration of the detector parameters used by the sensors in the process of cleaning raw measurement data based on previously acquired knowledge about the monitored phenomena, and then updating them regularly during the fusion of data from multiple sensors in subsequent cycles. The updated detector parameters can then be transmitted back to the previously installed measurement sensors (when their communication module enables the receiving of data from the UAV nomadic gateway) or used in the software of the next-generation (improved) sensors.

#### 2.4.1. Initial Version of the Anomaly Detector Software

**Figure 2:**
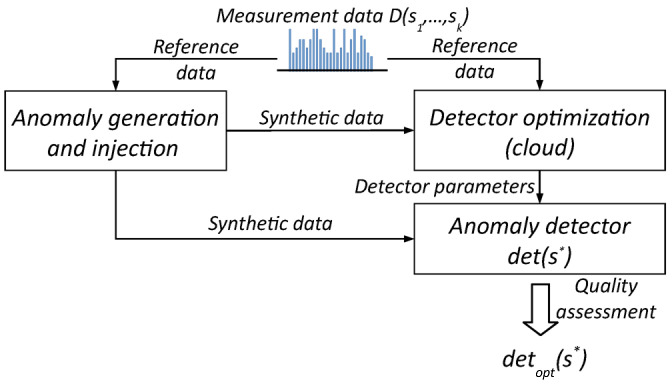
Data-free scheme for development of the anomaly detection software.

Development of the initial version of the anomaly detector software requires knowledge of the design of the measuring end device, methods of measuring selected soil parameters and, as argued earlier, the physical properties of the monitored processes. When we can formally characterize individual anomalies by a set of measurable parameters, for example, as shown earlier in Figure 1, the first step of software development for the detector should be determining the optimal values of these parameters. This, in turn, requires a sufficiently large volume of raw measurement data. If we do not have such a set, it may be helpful to use the Data Free scheme presented in Figure 2, which uses the synthetic data mentioned earlier.

We implemented this scheme in our CLaaS technology stack on the TASKcloud [16] computing cloud by adapting it from a portfolio of federated learning models [18]. The computationally advanced “teacher” module searches for optimal parameter values for individual anomalies, while the “student” module is the actual anomaly detector software to be installed on the end device. Both modules—the “teacher” and the “student”—are fed with data containing anomalies, and the entire process continues until the “student” reaches the desired level of detection quality. The result of this operation is a set of detector parameters $det_{opt}\left(s^{*}\right)$, which is the same for each sensor $s^{*}\in s_{1},\ldots ,s_{k}$. The “teacher” module can use various models: in [15], we described the solution we implemented using *simulated annealing* (SA) to optimize the detector parameters, whereas three alternative solutions using neural models are further detailed in Section 3.

#### 2.4.2. Continuous Development of the Anomaly Detector Software

**Figure 3:**
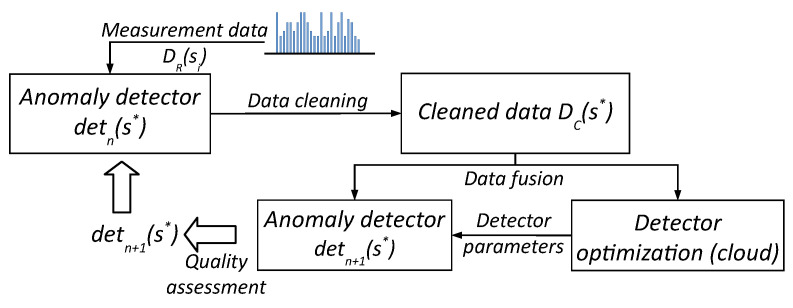
Life-Long Learning scheme for development of the anomaly detection software.

During regular exploitation of the Rural IoT ecosystem sensors over a long time period, the volume of cleaned data $D_{C}\left(s^{*}\right)$ collected from sensors $s_{1},\ldots ,s_{k}$ systematically increases. These data can then be used for continuous evaluation of the quality of the current version of the $det_{n}\left(s^{*}\right)$ detector software installed in the sensors, through comparison with the respective raw data $D_{R}\left(s_{i}\right)$, i=1,…,k. The Life-Long Learning federated learning scheme [18] is suitable for this purpose, which we also adapted to our CLaaS technology stack, as presented in Figure 3.

All measurement data collected to date in our TASK cloud instance are delivered to the input of the module that implements a selected ML model, which is systematically fine-tuned with the $D_{C}\left(s^{*}\right)$ data. As a result of this process, a set of new detector parameter values (e.g., neural network weights), $det_{n+1}\left(s^{*}\right)$, is determined. If the results of anomaly detection obtained for the newer version are better than the results obtained so far, the new set of parameters $det_{n+1}\left(s^{*}\right)$ is, if possible, sent back to the existing sensors $s_{1},\ldots ,s_{k}$ of the ecosystem as an update to their software, or is set in the software of all newly installed sensors. In both cases, involving the Data Free and Life-Long Learning schemes, the quality of anomaly detection is assessed with respect to reference signals, as described later in Section 4.

## 3. Neural Networks for Time-Series Segmentation

Anomaly detection in measurement data time-series using neural networks provides a number of advantages that have been repeatedly confirmed in the literature [19]. Neural networks are powerful tools that can learn relevant features from data, adapt to the different characteristics of time-series, and learn optimal segmentation points, making them more robust than traditional methods for handling noisy or incomplete data. These models, however, come at a cost. First, in order for neural networks to learn efficiently, they generally require a lot of training data. Second, they can be computationally costly to train and deploy, particularly on end devices with limited resources. Finally, it can be difficult to comprehend how they make decisions regarding specific types of anomalies. While the latter task appears to be relatively straightforward once there is a comprehensive model explaining the physics of the process producing the data to be analyzed, the first two need a more thorough investigation. For this purpose, we build upon the approach we adopted earlier in [15].

With the limited set of real measurement data that we had at our disposal, we first constructed reference signals based on our understanding of the originally recorded time-series data. All explainable anomalies shown in Figure 1 were corrected by smoothing them out automatically or replacing them with sequences of samples from the nearest preceding or following days, clearly marked by PV jumps between two consecutive sunrises. We then generated hundreds of mutated time-series by injecting various anomalies that had been previously identified into the reference series, with the values of individual parameters varying randomly. Anomalies were introduced realistically, namely, for each anomaly and signal type, a randomly selected week was subject to local mutations during one of its days. The data obtained in this way, although synthetic, contained anomalies that were suitably related to the physical properties of the measurement processes.

In turn, to address the limitations of constrained measurement devices at the edge, we took a closer look at alternative types of neural networks for anomaly detection to find a reasonable compromise between the time complexity of the underlying process and the accuracy of signal segmentation. We conducted comparative experiments based on the following three approaches:

- *Neural-primed heuristic (NPH)* using two pre-trained neural models to detect the center and edges of anomalies and heuristics to determine the extent of anomalies.
- *Autoencoder-based (AEB)* comprising a simple 5-layer autoencoder architecture without skip connections but, instead of reproducing the input, it outputs the anomaly probabilities for each signal sample.
- *U-Net-based (UNB)* using the original U-Net with a 2D input [20] reduced to a number of time steps corresponding to the length of a 1D signal, and with 1D convolutions maintaining the concept of skip connections, i.e., connecting the outputs of the encoder layer to the inputs of the decoder layer at the corresponding resolutions.

The reason for considering three different anomaly detector variants with different complexities and different structures was the need to achieve a compromise between accuracy and processing time, which is important when using constrained end devices. Therefore, it is worth investigating which variant offers the best compromise between detection accuracy and processing time, or, at least, to be certain of the choice in the case that one of the variants dominates over the others.

### 3.1. Signal Normalization

Normalization can be understood as a simple transformation of a signal to reduce some of its variance and make the task of detecting anomalies easier. The lack of normalization would result in the need to use much more data for training to make them more representative. Two types of normalization of signal samples were tested:

- Dividing by the mean, i.e., $x_{N}=x/\mu_{N}$, where $x_{N}$ is the normalized value of a signal sample and $\mu_{N}$ is the mean value of samples in some fragment *N* of the signal sample.
- Normalizing by the average and standard deviation, i.e.,  $x_{N}=\left(x-\mu_{N}\right)/\sigma_{N}$, where $\sigma_{N}$ is a standard deviation of a signal sample’s values in some range *N* of the signal sample.

Both types of normalization were tested for a global variant (i.e., the entire signal $N=N_{max}$) and a local one (i.e., the signal portion $N<N_{max}$), and fed to the neural networks as input. All nine combinations of normalization or its absence were checked. The best solution was to adopt only local normalization by the mean.

If an anomaly has a constant amplitude, global normalization introduces additional variance, thus making it more difficult to detect. Normalizing by the mean and standard deviation can flatten out some anomalies, such as instabilities, which are characterized by an increased standard deviation relative to the rest of the signal.

### 3.2. Neural-Primed Heuristic (NPH) Approach

The neural-primed heuristic approach combines heuristic rules with neural networks, for example, by integrating statistical thresholds with deep representations to improve precision and adaptability. This hybrid idea underlies notable systems such as AlphaGo [21], which merges neural evaluation with Monte Carlo Tree Search, and IBM’s Neuro-Symbolic Concept Learner [22], which couples neural perception with symbolic reasoning.

In our application, the approach comprises two stages. First, neural models are trained to detect the center and edges of individual anomalies. In the second stage, based on the probabilities of occurrence of anomaly centers and edges obtained in the first stage, the anomalies are segmented using a heuristic method. For both stages, the same signals from training subset are used.

#### 3.2.1. Training Stage of NPH

The neural network models were trained based on training examples containing input vectors in the form of fixed-length signal fragments. These examples can be positive (i.e., containing an anomaly) or negative (i.e., without any anomaly).

Two types of model were trained with respect to the locations of anomalies within the input vectors:

- ANN-Center uses vectors with an anomaly located in the middle of the input vector as positive examples;
- ANN-Edge uses vectors with left anomaly boundary in the middle of the input vector as positive examples.

In this way, eight models were trained: two for each of the four anomaly types shown in Figure 1. Each model returns the probability of the anomaly center or its left edge occurring in a given signal fragment. The probability of the right edge occurring is determined by applying the input signal with samples in reversed order to the ANN-Edge  model.

In our experiments, the structure of the neural network was the same for the ANN-Center and ANN-Edge models except for the number of inputs, which slightly differed for each of the models and also depended on the anomaly type. Several structures with different numbers of Conv1D convolutional layers and fully connected layers were tested.

Ultimately, a four-layer structure consisting of two convolutional layers and two fully connected layers was selected. The convolutional layers contained 16 filters with a mask width of 5 and 8 filters with a mask width of 3, respectively. The fully connected layers consisted of 20 and 2 neurons, respectively. The first three layers used the ReLU activation function, while the last layer used the softmax activation function to obtain a measure of the probability of anomaly occurrence for a given signal sample. The loss function was cross-entropy and the optimization algorithm was the adaptive moment method ADAM [23].

One of the key problems we faced was selection of the length of the signal fragment *N*, constituting the length of the input vector fed to the network. Longer signal fragments allow for better highlighting of anomaly features against the background of the correct signal but are associated with a longer data processing time by the neural network, which is roughly proportional to the length of the input vector; this is crucial to take into consideration when processing signal data using an MCU. Longer signal fragments can also result in over-fitting to the training data. Therefore, a reasonable compromise between the measure of segmentation correctness and data processing time is necessary.

Based on the results presented in Table 2, the signal length *N* for detecting the center of the anomaly was chosen to be equal to three average anomaly widths, and that for detecting the edge was equal to two average anomaly widths.

The numbers in the Input Vector Length row indicate the mean anomaly length multipliers that provide input lengths for ANN-Center and ANN-Edge models; e.g., 3/2 for anomaly peak with 2.89 mean length gives a 3·2.89≅9 sample vector as the ANN-Center model input length and 2·2.89≅6 as the ANN-Edge model input length.

After analyzing the results presented in Table 2, we decided to use 3/2 multipliers due to their much better results compared with 2/1.5 multipliers. When longer input vectors were used, the results were slightly better for some anomalies, but at a much higher computational cost. The input vector lengths for particular anomalies are presented in Table 3.

The number of multiplications needed to classify a single sample results from the network architecture and is equal to L=5·16·N+3·16·8·N+8·20·N+20·2=624·N+40, where *N* is the number of samples of the input vector, which depends on the model type and anomaly type, as presented in Table 3. The total number of multiplications to compute probability vectors for heuristic anomaly detection per signal sample can thus be calculated using

(3) $$L_{det}^{NPH}=\;\sum_{*}L_{class}^{*,center}+2\cdot L_{class}^{*,edge}=632800,$$

where ∗∈{P,B,J,I}, $L_{class}^{*,center}$ denotes the number of multiplications to classify the center of anomaly “∗” for a single sample, and $L_{class}^{*,edge}$ is the number of multiplications needed to classify an edge of anomaly “∗”, respectively.

#### 3.2.2. Heuristic Segmentation of Anomalies

In the second stage of NPH, the neural network models are used to detect anomalies in the signal by feeding a signal window of fixed length to the input of each model. The anomaly center and left edge probabilities are obtained directly from the ANN-Center and ANN-Edge models, while the right edge is obtained by inverting the input vector to the ANN-Edge model. The overall NPH anomaly segmentation process is described in Algorithm 1.
**Algorithm 1** Anomaly segmentation via the NPH method.1:**function** AnomSegmentationByNPH ($signal,ann_{center},ann_{edge},th_{region},th_{h}$)2:    ▹ **Input parameters:**3:            ▹signal—time series4:            ▹$ann_{center},ann_{edge}$—neural models for center and edge of anomaly detection5:            ▹$th_{region}$—threshold for a region of maximum6:            ▹$th_{h}$—threshold for center or edge maximum value acceptation7:    ▹ **Output:**8:            ▹anoms—a set of anomaly regions** **9:      $probs_{c}\leftarrow$CalcProbs(signal, $ann_{center}$)                               ▹ anomaly probabilty calculation10:**    **$probs_{l}\leftarrow$CalcProbs(signal, $ann_{edge}$)11:**    **$probs_{r}\leftarrow$CalcProbsRevers(signal, $ann_{edge}$)                            ▹ using signal in reverse order12:    **for all** keypoint∈{c,l,r} **do**        ▹ center and left and right edges of anomaly region13:        $maxima_{keypoint}\leftarrow$MaximaDeterm($probs_{keypoint}$,$th_{region},th_{h}$)14:    **end for**15:    anoms←PotentialAnomDetermination($maxima_{c},maxima_{l},maxima_{r}$)16:    grades←GradesCalculation(anoms)            ▹ grade calculation using Formula (4)17:    anoms←OverlappedAnomRemoval(anoms,grades)18:    **return** anoms19:**end function**

**Figure 4:**
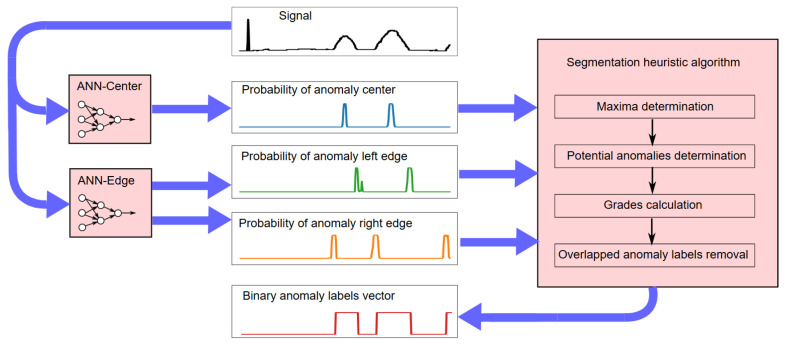
NPH processing scheme.

The NPH processing scheme is shown in Figure 4. Blue arrows indicate the direction of data flow.

A signal fragment $N_{*}$ is considered a potential anomaly when it has a sufficiently high maximum probability for the left edge of an anomaly on its left side $N_{*}^{L}$, a sufficiently high maximum probability of the right edge of an anomaly on its right side $N_{*}^{R}$, and a sufficiently high maximum probability of a center anomaly near its center. For simplicity, a single threshold $th_{h}$ value is assumed for all three maxima, after which the given maximum is taken into account for segmentation. The allowable ranges for the widths of individual anomalies were also assumed to be equal to the minimum and maximum widths of anomalies of a given type determined from the training data. Determining values of the local maximum probability involves adopting a threshold $th_{region}$ for the height of the maximum region, which may contain only one maximum (analogous to the peak prominence as a feature of mountain peaks, which is the elevation of the lowest contour within which there is no higher peak).

**Figure 5:**
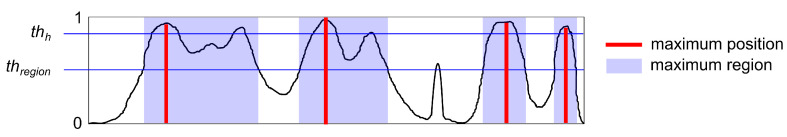
A sample anomaly probability chart with maxima and regions determined via procedure MaximaDeterm.

The regions and chosen maxima are shown in Figure 5 as an exemplary anomaly probability chart.

**Figure 6:**
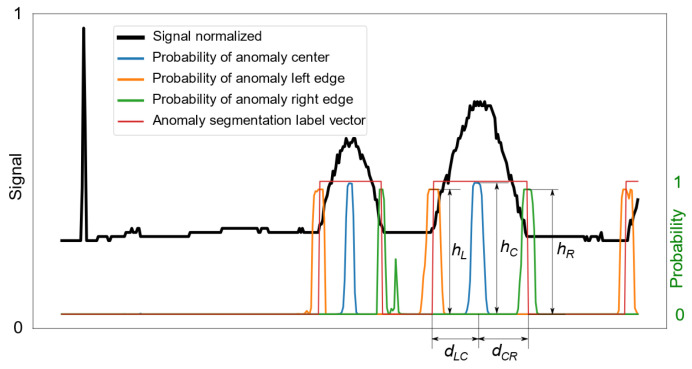
A sample waveform of a signal containing a bump, along with the anomaly probabilities for the center and edges.

Given maxima for all key points of the anomaly region, using the PotentialAnomDetermination procedure, we can generate potential anomalies from triplets of maxima occurring in the appropriate order, with the width of the potential anomaly limited to the maximum anomaly width in the training set. It is assumed that anomalies of the same type cannot overlap within one measurement signal. Therefore, to assess the significance of a potential anomaly, the following metric was defined:

(4) $$grade=h_{L}h_{C}h_{R}f_{d}\;,$$

where $h_{L}$, $h_{C}$, and $h_{R}$ denote the values of the respective probability maxima in Figure 6, whereas $f_{d}=\min\left(d_{LC},d_{CR}\right)/\max\left(d_{LC},d_{CR}\right)$ is a measure of the centrality of the maximum probability of the anomaly center relative to the maxima of the probabilities of the left and right anomaly edges. If potential anomalies overlap, those with lower grade are discarded, as specified in Algorithm 2.
**Algorithm 2** Overlapped anomaly removal for NPH detection.1: **function** OverlappedAnomRemoval(anoms,grades)2:    ▹ **Input parameters:**3:            ▹anoms—set of potential anomalies represented of regions of signal4:            ▹grades—grades for all potential anomalies calculated by Formula (4)5:    ▹ **Output:**6:            ▹anoms—a set of the most reliable and not overlapped anomalies**  **7:    **for** n=1,…,K **do**              ▹*K*—the number of potential anomalies8:        **for** m=1,…,K,n≠m **do**9:             **if** Overlapped(anoms[n],anoms[m])  **and**grade[n]≤grade[m] **then**10:                 MarkForRemove(anom[n])11:           **end if**12:        **end for**13:    **end for**14:    anoms←RemoveAllMarkedAnoms(anoms)15:    **return** anoms16:**end function**

An example signal waveform, along with the associated detection results, is shown in Figure 6. In a situation where there is a possibility of anomalies overlapping, some of them are rejected based on evaluation of the grade measure defined in Equation (4), which depends on the product of the heights of the maxima and the degree to which the center lies in the middle of the interval between the left and right edges.

The entire heuristic stage of NPH, after determining the probability vectors of the anomaly center and edges, is repeated for different values of the threshold $th_{h}$ using the training data to determine its optimal value.

#### 3.2.3. Cyclic Training and Generation of Negative Examples

**Figure 7:**
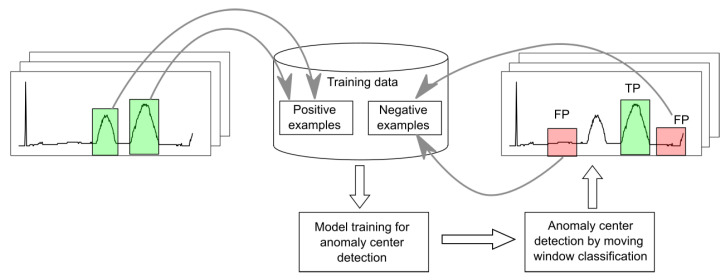
Training and detection cycle with the addition of difficult negative examples.

For each anomaly type, the learning and detection processes in either stage of NPH are performed repeatedly. In the first round, positive examples with anomalies and randomly selected negative examples without anomalies are extracted from the training data and used to train the models. Then, after each round, difficult negative examples are selected based on the detection results. These are signal fragments falsely classified as positive with a high probability of containing anomalies, as illustrated in Figure 7.

Using these false positive (FP) examples to train models in the next round allows for a significant reduction in the false positive rate (FPR). A motivation for this process is the inability to check before the first cycle, in which correct parts of the signal may be confused with anomalies. From cycle to cycle, increasingly difficult false positive examples are extracted, making it increasingly possible to distinguish true anomalies from apparent ones.

### 3.3. Autoencoder-Based (AEB) Approach

There are numerous examples in the literature of successful applications using autoencoders to detect anomalies in time series, particularly in approaches that treat anomaly detection as a sequence labeling task. For example, in [24], attention mechanisms and autoencoders were combined to capture both local and long-term relationships in time-series data. Other approaches take advantage of regularizing autoencoders; for example, through the use of wavelet transforms [25]. However, few studies have focused on pinpointing the exact time steps in which anomalies occur [2].

An alternative approach, which was applied in our project for delimitation of the anomalies shown in Figure 1, is to use an autoencoder that outputs an anomaly probability value for each input signal sample. The most important feature of such a neural network is that it has the same number of outputs as inputs.

**Figure 8:**
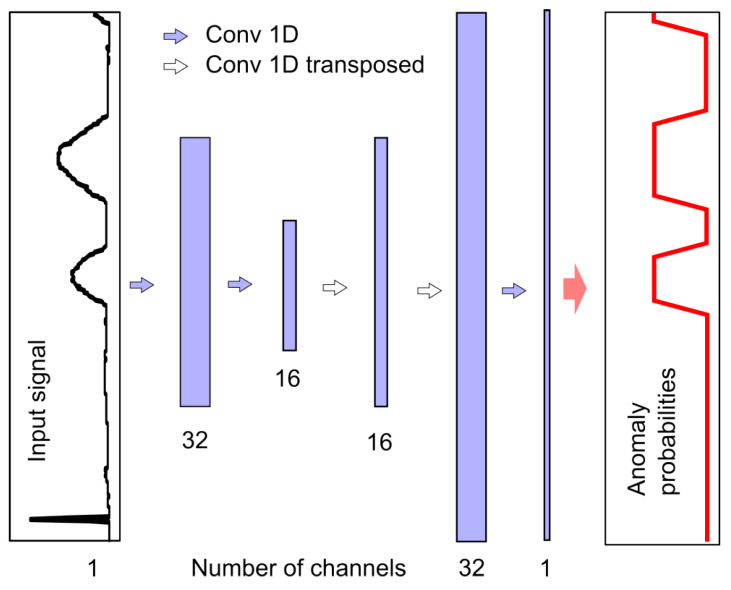
AEB model structure.

To achieve this, one can use many one-dimensional convolutional layers of the *Conv1D* type, which do not cause shortening of the signal at the output of each layer. Another possibility may be the use of layers with padding parameter set to same and the strides parameter set to 1. Another is shortening the output signal in the initial network structure (e.g., using strides=2), and then extending the signal using transposed convolutional Conv1DTranspose layers in the final part of the network; this latter solution was adopted in this study, as outlined in Figure 8.

By compressing information in the central part of the structure, this type of network allows for overall analysis of the entire input signal fragment with a small number of layers, which is important when considering segmentation problems. More details regarding this type of layer may be found in [26].

The number of channels obtained after filtering is indicated at the bottom of Figure 8. Each filter corresponds to one output channel, and we used filters with a mask of length 7. The sample value in the output channel is calculated using the ReLU activation function on the weighted sums of seven-sample neighborhoods of samples from individual input channels. The exception is the last layer, which contains only one filter and a linear activation function. Values below zero are converted to zero and values above 1 are converted to 1, such that each output value corresponds to the probability of an anomaly occurring. When using AEB, similarly to NPH, a separate model was created for each pair (i.e., an anomaly and a physical parameter), resulting in a total of 12 models that were trained and tested independently. The number of multiplications per sample for one model is equal to 5679. Taking into account that four models are needed to detect all anomalies, the number of multiplications per sample is thus $L_{det}^{AEB}=22,716$. The number of additions is almost the same.

#### 3.3.1. AEB Training

The network was trained with signal fragments of a fixed length equal to 30 times the average anomaly length; each randomly selected fragment contained several labeled anomalies at random locations. Such a signal fragment length guarantees an appropriate amount of background signal for anomaly isolation. The input length for an autoencoder network increases the training time but has no effect on the detection time, provided that the network structure contains only convolutional layers. However, for longer signal fragments, no improvement in results was observed. An additional problem in the case of using convolutional layers in the encoder is the reduction of the signal resolution in individual layers (usually twice) due to subsampling (e.g., *stride* = 2, or using the *MaxPool1D* operation in the case of UNB, as discussed below). As it may be a good idea to set the input length as a power of 2, in this study, we rounded this value up to the nearest power of 2. In Table 4, the average lengths of anomalies of the respective types and the determined lengths of signal fragments fed to the autoencoder as input are presented.

For each anomaly that occurred in the training data signals, 50 randomly selected fragments containing the anomaly were generated to train the autoencoder in a way that is invariant with respect to the position of the anomaly in the analyzed signal fragment. The training set was supplemented with 500 randomly selected signal fragments containing mainly the background, thus being ten times greater than the number of fragments that definitely included anomalies.

For each pair (an anomaly and a physical parameter), the loss function was the mean squared error (MSE), calculated as the squared difference between the probabilities output by the network and the ground truth labels. Training was performed for 100 epochs, with modification of the network weights using the ADAM method mentioned above. In each epoch, all training examples were grouped into minibatches.

#### 3.3.2. Detection of Anomaly by AEB

**Figure 9:**
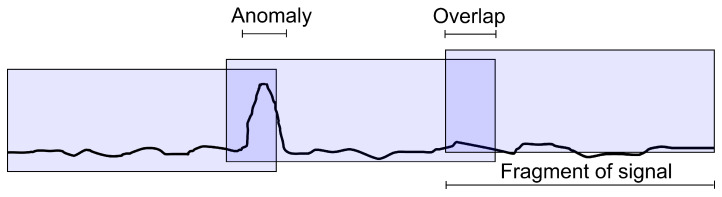
Sliding window scheme for AEB and UNB anomaly segmentation.

Segmentation of anomalies into T, M and pH signals required feeding the model subsequent signal fragments with lengths depending on the type of detected anomaly specified in Table 4 as the input. Each subsequent fragment overlapped the previous one by about 30%, as shown in Figure 9.

The model output represents the probability of an anomaly occurring in each sample of the selected signal fragment. In overlapping areas, when a given sample belongs to two independently processed signal fragments, the anomaly probability is calculated as the maximum value of the two obtained values. A 30% overlap allows the anomaly context to be included if one of the fragments contains only a part of it, as illustrated in Figure 9. Higher overlap values increase the time complexity of the detection process, but do not significantly improve the results.

The final binary anomaly labels are determined by thresholding the probability values. The standard threshold is 0.5, above which the sample value is considered anomalous. However, these results can be improved by determining an optimal threshold for the selected criterion (e.g., intersection over union (IOU), as defined in Equation (1)) for each neural network model. Such a threshold can be determined using the aggregated probabilities of anomaly occurrence in individual samples for all signals in the training set, as shown in Figure 9. Computational efficiency may be ensured by concatenating probability vectors into one long vector, then using Algorithm 3 to sort it in ascending order, and finally calculating the IOU for each subsequent probability value by counting the true positive labels.
**Algorithm 3** Probability threshold selection for IOU maximization.1:**function** OptimalThresholdSelection(pr,t)2:    ▹ **Input parameters:**3:       ▹pr—aggregated vector of anomaly probablities for all training signals4:       ▹*t*—aggregated vector of true labels for all training signals5:    ▹ **Output:**6:       ▹$th_{IOUmax}$—optimal threshold for IOU measure**  **7:    L←length(*t*)8:    P←|t|                ▹ number of anomaly (positive) samples9:    IOUmax=P/L10:  pr←sort(pr)11:  t←t[argsort(pr)]             ▹*t* is sorted in the same order as pr12:  TP←P                 ▹ initial number of true positive samples13:  FP←L−P                 ▹ initial number of false positive samples14:  **for** n=1,…,L **do**15:        TP←TP−t[n]16:        FP←FP−(1−t[n])17:        IOU←TP/(P+FP)18:        **if** IOUmax<IOU **then**19:              IOUmax←IOU20:              $th_{IOUmax}\leftarrow pr\left[n\right]$21:        **end if**22:    **end for**23:    **return** $th_{IOUmax}$24:**end function**

### 3.4. U-Net-Based (UNB) Approach

Originally developed for 2D biomedical segmentation [20], U-Nets can be adapted to 1D time series, enabling the segmentation of anomalies or events in temporal fragments. Such models have shown versatility across domains such as sleep staging [27], activity recognition [28], and anomaly detection [3]. Their compact design yields benefits on small datasets; however, compared with autoencoders, they demand more memory and energy due to deeper layers and skip connections, making them better suited for cloud deployment.

**Figure 10:**
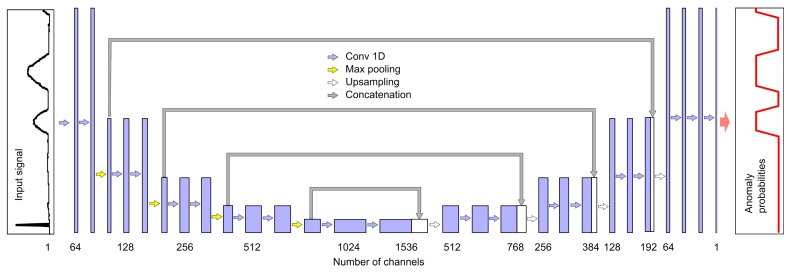
U-Net structure.

This approach differs from AEB only in the structure of the network used. All other aspects of training and detection were the same. Details of the UNB structure are shown in Figure 10.

As in AEB, the network consists only of convolutional layers—in this case, a total of 23 layers—that are considered to be parts of the structure that process data using weight parameters. In Figure 10, the layers are indicated by blue and white arrows. Blue arrows indicate filtering of the signal by the convolution function *Conv1D* to an output signal with the same resolution. The width of all *Conv1D* filters is equal to 3. In the encoder part, the signal resolution is reduced using the *MaxPool1D* operation every few layers, as indicated by the yellow arrows. This resolution reduction is compensated for by an increase in the number of filters and, therefore, channels. In the decoder part, the resolution is increased using the *Conv1DTranspose* function with filter width equal to 2. Unlike AEB, UNB has additional connections, namely, skip connections, as indicated by grey arrows. These allow for the addition of signals obtained in much earlier stages of processing (i.e., in the encoder part) to the decoder part.

The number of multiplications per sample for one model is equal to 1,302,413. Taking into account that four models are needed to detect all anomalies, the number of multiplications per sample is $L_{det}^{AEB}=5,209,632$; this is about 230 times more than that required for the AEB model.

## 4. Experiment

In this section we provide the details of the performed experiment, including the environment setup, performance metrics, analysis of results, and discussion.

### 4.1. Environment Setup and Data

For training and testing the NPH, AEB, and UNB methods, the same data were used as in the case of evaluation of the heuristic method with simulated annealing optimization described in the work [15]. The data consisted of real signals obtained from sensors placed in the soil with different variants of trends and with added artificially generated anomalies, namely, peaks *P*, bumps *B*, jumps *J*, and instabilities *I* of different widths and amplitudes inserted in random places. Details regarding the generated data can be found in [15]. Experiments focused on the NPH, AEB, and UNB methods were performed independently for each anomaly type {P,B,J,I} and each physical parameter T, M and pH, with 4·3=12 model training and testing experiments in total. Neural network models were trained using our cloud computing facility and one *Nvidia H100* card. Training was performed using the Python 3.11.7 interpreter and the Tensorflow 2.18.0 library. The source code for training and testing all described methods is available from https://ieee-dataport.org/documents/rural-iot-soil-data (accessed on 21 August 2025).

Details on the neural network structure, optimization method, and loss function are described in Section 3.2 for the NPH method, Section 3.3 for the AEB method, and Section 3.4 for the UNB method.

**Figure 11:**
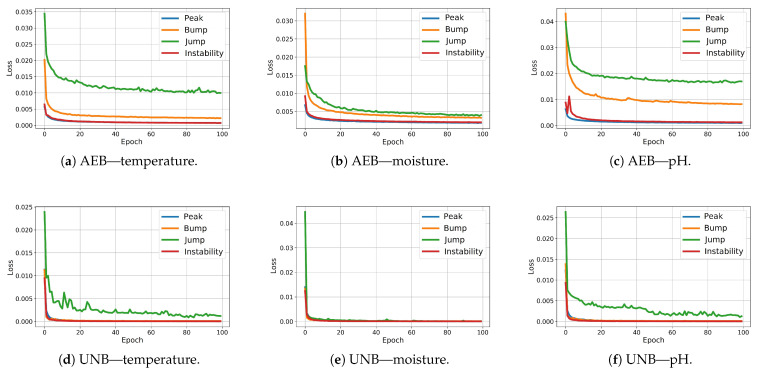
Loss diagrams for AEB and UNB model training.

Figure 11 shows the MSE loss function plots obtained during training for the AEB and UNB models. In the case of NPH, for each pair (anomaly, physical parameter), two models are required to detect the center and edge of the anomaly.

### 4.2. Results

The test error values obtained for the NPH method described in Section 3.2 are presented in Table 5. They were calculated for the test data not used in the detector construction process, as described in Section 2.2.

These results were obtained for specific threshold values, based on which detectors decided whether a given signal fragment was, in fact, an anomaly. By manipulating the values of these thresholds, we can obtain a trade-off between the FPR and FNR errors described in Section 2.3. Lower threshold values result in reduced FNR and increased FPS values, and vice versa. In this case, the quality measure of the detection system, regardless of the adopted detection threshold, can be considered as the area under the ROC curve (AUC), when the coordinate axes are FPR and FNR, and the tolerance threshold (or a combination thereof) is the parameter.

The values of the test error obtained for the AEB method described in Section 3.3 are presented in Table 6, and those for the UNB method described in Section 3.4 are presented in Table 7.

Comparing the test FPR and FNR error values and the IOU rates for the NPH, AEB, and UNB models presented in Table 5, Table 6 and Table 7, respectively, it can be seen that the error values were significantly smaller for the AEB and UNB methods, whereas the IOU rates were higher; however, this does not necessarily mean that these methods are better than the NPH for anomaly detection on end devices. It is equally important to consider the processing power demand of the end device, the amount of data available for training, and the level of support from the cloud instance for continuous improvement of the model parameters. The weaker results obtained for the NPH method compared with the AEB and UNB methods are probably due to the quality of the heuristics, which were not optimized. On the other hand, heuristics based on human knowledge of the problem may allow for dealing with the problem of small datasets, with which training autoencoders can be difficult. Nevertheless, the results of anomaly segmentation alone with the NPH method were significantly better than those with the SA method. The average IOU value for the test data for the SA method was 0.25, which was almost twice as bad than the value obtained with the NPH method (0.48), as presented in Table 8. The AEB and UNB methods both present certain advantages and disadvantages. For example, one advantage of the latter is its higher prediction accuracy, as confirmed by our test results, while its disadvantage is a higher power demand due to its more extensive computational structure in comparison with the AEB, which may be a crucial consideration for a constrained device. Table 9 presents the AUC values obtained for both AEB and UNB methods. These values are presented only for these two methods as, in the case of NPH, the anomaly probabilities were not determined for individual samples due to the heuristic nature of this method.

**Figure 12:**
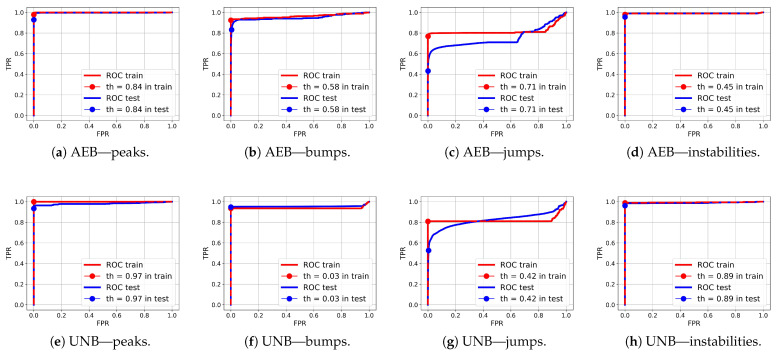
ROC curves for anomaly segmentation in temperature signals for the AEB and UNB methods.

**Figure 13:**
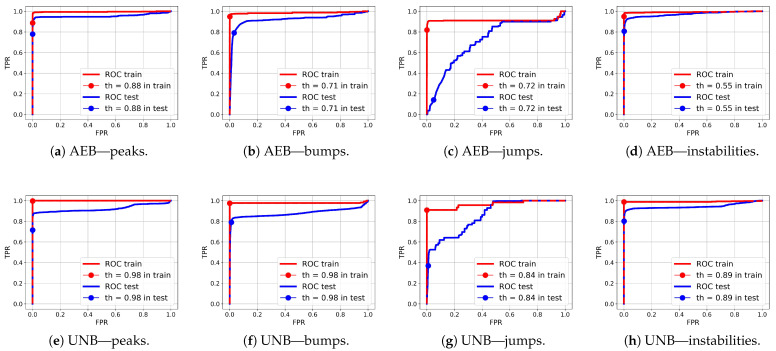
ROC curves for anomaly segmentation in moisture signals for the AEB and UNB methods.

**Figure 14:**
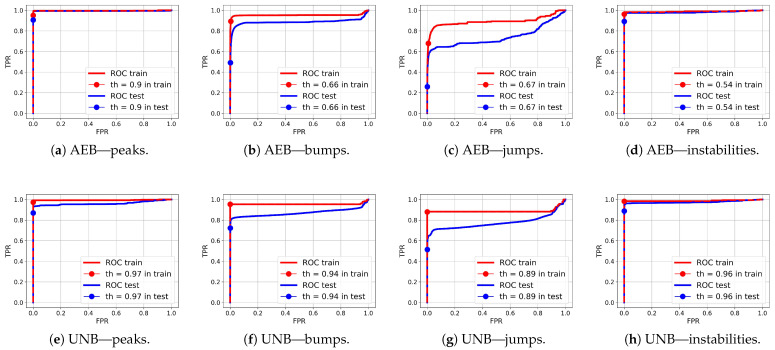
ROC curves for anomaly segmentation in pH signals for the AEB and UNB methods.

ROC curves for anomaly segmentation in temperature (T) signals are presented in Figure 12, in moisture (M) signals in Figure 13, and in pH signals in Figure 14.

**Figure 15:**
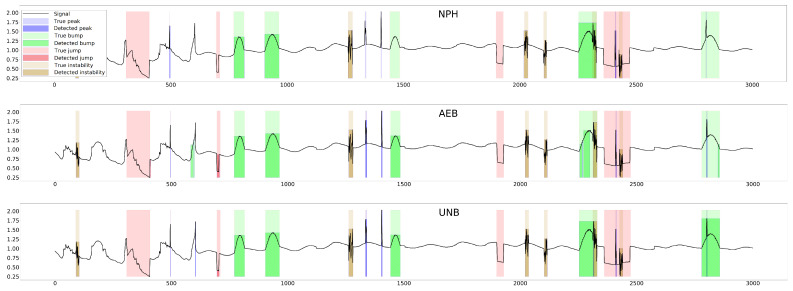
True and detected anomalies in temperature signal from sensor s22.

**Figure 16:**
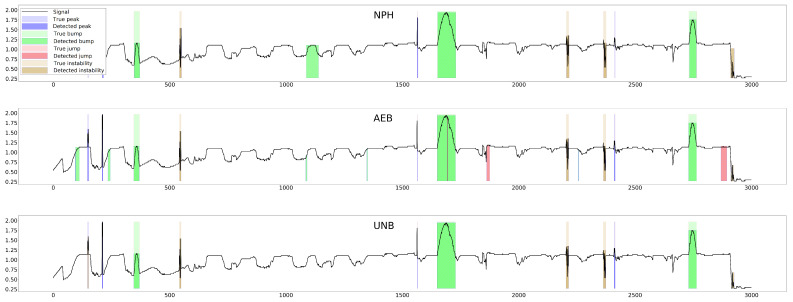
True and detected anomalies in moisture signal from sensor s22.

**Figure 17:**
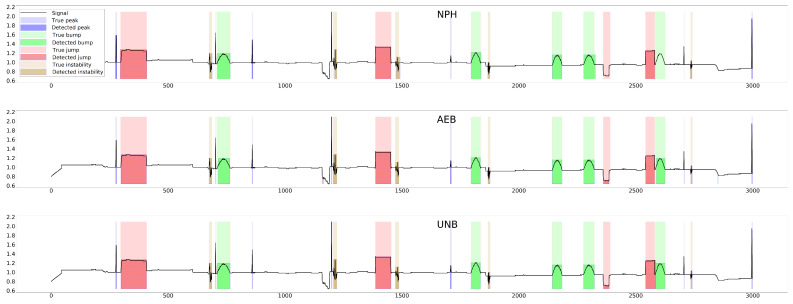
True and detected anomalies in pH signal from sensor s22.

The parameter of each curve is the anomaly probability threshold, the values of which start at 0.0 in the upper right corner and end at 1.0 in the lower-left corner. The red color indicates the curve for the training data (sensors $S_{tr}=\left\{s01,s02,s23\right\}$), and blue for the test data (sensors $S_{te}=\left\{s03,s10,s21,s22\right\}$). The circle indicates the place on the curve corresponding to the threshold $th_{IOUmax}$, giving the maximum IOU value for training data obtained by Algorithm 3. Figure 15, Figure 16 and Figure 17 show plots of T, M, and pH signals from sensor s22 and the regions of true and detected anomalies determined by the three methods used in this work. Signals from this sensor were used only for testing, and thus, the results indicate the generalization ability of the models. The results and graphs for the signals used for training of models are better and more accurate.

### 4.3. Discussion

All of the previously discussed models for detecting and segmenting anomalies in the analyzed signal fragments, after appropriate optimization/tuning on the cloud instance (performed according to the schemes depicted in Figure 2 and Figure 3) and their conversion from Python to C, were integrated with the sensor’s software to implement a daily (every 24 h) cleaning cycle, determined according to two consecutive leading edges of the PV signal. Measurements of the values of individual T, M, and pH signals are carried out with different sampling periods, depending on the dynamics of each individual signal. In the current implementation of our sensors, this period is approx. 10 min for all signals. After the cleaning process, the sample values of individual signals can be aggregated over longer time intervals without losing information, to a maximum of 70 min for M, 90 min for T and PV, and 220 min for pH. This aggregation is necessary to maintain the byte payload of data to be sent to the UAV at an appropriately low level, resulting from the specifications of the LoRaWAN technology (up to 250 bytes per single packet in Europe) [13].

The following steps briefly summarize our daily cleaning cycle, as previously specified in detail in [15]:

1. Samples delayed due to a power gap may have valid values, so they are preserved but marked as “misplaced” for further processing in the cloud.
2. All “absolute error” samples are set to the “empty” value.
3. Values of samples labeled by the anomaly detector as “peak” and “jump” are interpolated with respect to their neighbors.
4. Samples labeled as “bump” are smoothed relative to the values of their adjacent fragments.
5. Finally, samples labeled as “instabilities” are replaced with the signal trend samples, calculated as a daily moving average.

The rationale behind these steps is to first eliminate samples which may affect signal smoothing (steps 1 and 2), before correcting outliers and the most abrupt change points (step 3), followed by less abrupt ones (step 4). Smoothing of values is necessary to eliminate bias that could be introduced during calculation over the values neighboring more gentle changes, such as bumps and instabilities (steps 4 and 5).

**Figure 18:**
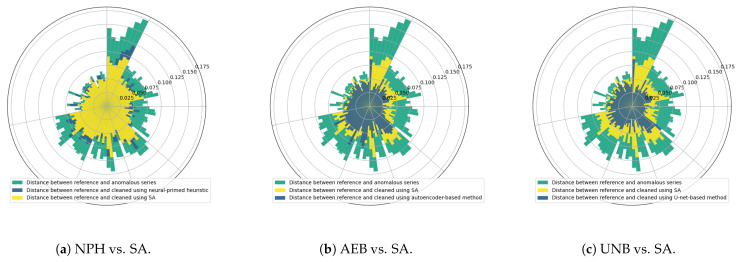
Comparison of effectiveness of time-series data cleaning using alternative detector models.

We evaluated the effectiveness of each of the previously analyzed anomaly detection methods defined in Figure 1 in the same way as in [15], that is, by measuring the average distances between each reference signal (ground truth) and the signals cleaned via the operations specified above. The polar charts shown in Figure 18 present the results we obtained for all seven (s01–s03, s10, s21–s23) devices, with distances averaged across 10 cleaned signals for every week of the lifetime of each sensor.

As a reference for assessing the effectiveness of time-series cleaning using detectors exploiting the neural network models NPH, AEB, and UNB, we used a heuristic method with simulated annealing-optimized (SA) detector parameters for each anomaly class specified in Figure 1. At least two time series were used for each signal (M, T, and pH): one with truly labeled anomalous samples, and another with samples labeled by the detector being optimized. Several independent optimization tasks were created in this way and were executed in parallel in the computing cloud, each targeting a specific combination of anomaly signal types. As a result, the best possible values of the parameters for individual anomalies were determined, such that each detected anomaly corresponded as closely as possible to the true anomalies [15].

The graph shows a relatively small improvement in signal quality with NPH over SA, whereas the improvements of AEB and UNB over SA are more significant. However, in the case of detection alone (without taking into account the effects of the cleaning algorithm), the NPH method was clearly better than the SA method, yielding almost twice the IOU value, evidence of which may be found in [14]. It also performed better than the AEB method in some cases (e.g., in segmenting bump anomalies in the moisture signal, as can be seen from Table 5 and Table 6).

The worst segmentation quality was observed for jump anomalies, as indicated by both the IOU values presented in Table 5, Table 6 and Table 7, as well as the AUC values presented in Table 9. The reason for this is likely that a jump anomaly involves a momentary change in the trend in the time series, while the trends in the signal outside of the jump appear normal, which can make the detection of such an anomaly difficult.

The best segmentation quality was achieved for the temperature signal. This is likely due to the greater predictability of the trend and its daily periodicity, which makes it easier to distinguish anomalies from random noise.

As shown in Table 9 and Figure 12, Figure 13 and Figure 14, the segmentation results for the training data were clearly better than those for the test data. This indicates that the test results can be improved through the use of larger amounts of training data.

The comparison of anomaly detection performance against processing time for the three methods is presented in Table 8. The intersection over union (IOU) measure was averaged across 12 detectors for combinations of four anomaly types and three measured physical parameters. It is also considered as a measure of generalization ability, as it was calculated only for the test sensors $S_{te}=\left\{s03,s10,s21,s22\right\}$. The number of addition operations in neural network model processing is approximately the same as the number of multiplications in each method and the same as the number of model parameters. Therefore, the total number of arithmetic operations can be approximately calculated by multiplying the number of multiplications by 2. Memory usage is also roughly proportional to the number of parameters of the neural model.

The last row of Table 8 provides the average time complexity, expressed as processing time (excluding the time required to load neural models from disk to working memory). These values are not directly proportional to the number of multiplications (neural network model parameters) due to other operations, such as input vector normalization, output vector summation, or heuristic segmentation in the case of the NPH method. Individual times were determined using one CPU and one GPU of an Nvidia H100 computer for segmentation scripts written in Python. However, in the target microcomputer environment equipped with measurement devices, C code is expected to be used. Given the hardware capabilities and different implementation languages, the processing times may be much longer and in different proportions for the individual methods. The best method for anomaly detection accuracy appears to be the U-Net-based (UNB) model. However, the large number of arithmetic operations required per signal sample can be problematic on a constrained device running an MCU. Therefore, the AEB method seems to be a reasonable choice, as it offers only slightly lower accuracy but at the cost of nearly 230 times lower processing time. On the other hand, while the NPH method appears to be dominated by the other two methods, it has potential for improvement (especially regarding the heuristic component).

Anomaly detection in sensor-generated time series is highly application-specific, and there is no universal method that is suitable for every scenario. In IoT and industrial contexts, models such as ARIMA, machine learning classifiers, clustering, and deep learning require tailored selection and tuning depending on the nature of the sensor data and the anomaly type [29]. For example, statistical and forecasting models turn out to be appropriate when time-series data exhibit clear trend and seasonal structures. An Isolation Forest scales well to high-dimensional, sparse, or unbalanced datasets. In environmental monitoring, such as high-frequency water quality sensors, researchers have found that combining regression-based methods, feature-based detection, and rule-based techniques improves performance, as each method excels at capturing different types of anomalies such as spikes, drift, or missing values [30].

Similarly, when comparing various deep learning frameworks, it has been highlighted that even state-of-the-art models (e.g., autoencoders, graph-based networks, LSTM encoder–decoders) cannot be universally applied across systems, as their effectiveness depends on many factors such as cross-sensor relations, temporal dependencies, and system-specific characteristics [31]. Autoencoders effectively adapt to complex, non-linear patterns [32]. Advanced multivariate deep models (e.g., InterFusion) are ideal for multi-sensor systems where both temporal patterns and cross-sensor dependencies matter [33]. GAN-based approaches such as TAD-GAN are powerful for anomaly detection, as they can jointly perform realistic time-series generation and discrimination, enabling subtle deviations from normal behavior to be effectively identified [34].

Consequently, the design of anomaly detection systems must be adapted to the specific application context, data characteristics, anomaly types, and operational requirements.

## 5. Conclusions

In this paper, we report the results of a three-year R&D project during which we developed and deployed a demonstration Rural IoT measurement ecosystem in a real operation environment. The proposed system enables the monitoring of soil parameters in vast areas, such as farmland, forests, or river banks, where there is no telecommunications infrastructure enabling the transmission of data from measurement sensors planted in the ground to a cloud instance for further processing. A key component of this ecosystem is a nomadic gateway carried by a UAV. While such a UAV may possess sufficient lifting capacity and operational range, the working conditions of the mobile gateway do not differ much from its stationary counterpart on the ground. As such, a number of problems relating to the use of ground sensors arise due to the moderate computational capabilities of their MCUs, battery capacity, and limited byte payload that can be sent from the sensor to a nearby UAV. The volume of data to be sent varies due to the timing of UAV flights, depending on the weather conditions and the availability of airspace over the monitored area. Hence, each sensor may be tasked to clean incorrect, redundant, or irrelevant samples in the collected raw data time-series, such that the ultimately transmitted packet is as small as possible and falls within the available limitations.

The cleaning of time-series data first requires detecting and locating anomalous samples which the sensor software can correct or remove to obtain cleaned data that are ready for sending. To address the problem of determining which specific signal samples the sensor software should classify as anomalous, we developed a method based on comprehensive interpretation of the physical characteristics of soil signal measurement processes, enabling us to distinguish four generic classes of anomalies. These classes can be parameterized, making it possible to explain and adjust the anomaly detection and segmentation processes to specific soil types, climates, or sensor modalities at any geographical latitude where agricultural activities are carried out. We tested four alternative solutions for anomaly detection: a solution with heuristically selected anomaly parameters and their SA-optimized variants [15]; NPH, which involves fully heuristic anomaly detection; and the more computationally expensive AEB and UNB models. We carried out a comparative assessment of these methods regarding their detection performance and computational complexity. We also validated their performance limits using sensors based on Arduino and LoRaWAN technology in realistic operational environments, including farms (54°07′12.0″ N 18°46′48.0″ E) and forests (54°0′50.0″ N 17°49′45.0″ E). It should be noted that, by ”realistic” we mean solutions that can be implemented using Arduino-compatible MCUs. Analysis of the time and memory complexity of the four tested models presented in this paper confirmed their satisfactory effectiveness in cleaning the anomalies defined in Figure 1 from the collected real-world time-series data.

Our experience from three growing seasons starting from 2023 indicates that sporadic power outages of individual sensors developed for the project, which were observed during irregular UAV operations caused by weather conditions (thus preventing UAVs from collecting data regularly on a 1- to 3-day basis), do not really pose a serious problem, and thus, the measured data can be quite effectively cleaned by the sensor and processed further in our TASKcloud instance [17]. However, further research is needed to determine operational limits for the production version of the measurement sensors, with further reduced battery capacity, PV cell, and box dimensions, compared with our current experimental version. The latter utilizes an Arduino-compatible 32-bit 64 MHz MCU with 256 KB of flash memory to store the executable code and 32 KB of RAM to store data, powered by a 350 mAh LiPo battery and a 135 × 165 mm PV cell with a nominal voltage of 6.0 V, all housed in a weather-resistant 175 × 225 × 80 mm box. At present, we are conducting a series of electrical measurement experiments to determine the sensor’s energy reserve limits, to make it more compact and sufficient for continuous operation over extended (weekly or monthly) periods. A detailed description of this experiment, however, is beyond the scope of the current paper. The ultimate goal of this research is to develop disposable sensors that can be dropped over large areas by UAVs, to further reduce the cost of deploying rural IoT networks.

## Figures and Tables

**Table 1:** Explainable deviations in soil signals.

Interpretation	Explanation	Type
Samples (values) stored in the sensor memory are shifted (delayed) in time relative to the original signal waveform.	When reading subsequent samples from probes placed in the soil by MCU, the system time counter stops due to a complete lack of power (e.g., after the battery has been discharged during night). Our constrained end devices do not have an autonomously powered system clock.	Power gaps
The value of a single signal sample falls outside the allowable range of its variability.	A short-term event occurred in the probe’s immediate vicinity, causing an erroneous reading of a given value.	Absolute error
The values of several adjacent signal samples remain within the correct range of signal variation, but significantly exceed the average values of the samples in both of their neighborhoods (left and right).	An event occurred in the probe’s immediate vicinity, which temporarily interfered with the read signal.	Peak
The values of several adjacent signal samples remain within the correct range of signal variation, but significantly exceed the average value of samples only in their left or right neighborhood.	During data reading, the state of the probe placed in the soil was permanently changed; e.g., by switching its power supply on or off (analog sensor) or resetting its reading register (digital sensor).	Jump
The values of signal samples in some sequence remain within a normal range of signal variability, but show a gentle and reversible deflection from its overall trend over a longer observation period (e.g., daily).	While reading subsequent samples, an event occurred that caused an observable, long-lasting change in the value of the measured parameter (e.g., flooding of the soil moisture probe).	Bump
The values of a certain sequence of samples oscillate with a small deviation from each other along the signal trend line.	When reading subsequent samples, the sensor power supply is unstable (e.g., when the battery is not sufficiently charged, the additional load imposed by MCU computations temporarily affects the PV cell output voltage).	Instabilities

**Table 2:** IOU rates for input vectors of different length when using NPH.

Input Vector Length	2/1.5		3/2		6/4		9/6	
	**Training**	**Test**	**Training**	**Test**	**Training**	**Test**	**Training**	**Test**
Peaks	0.16	0.04	0.39	0.33	0.56	0.43	0.55	0.54
Bumps	0.89	0.63	0.9	0.63	0.8	0.59	0.74	0.55
Jumps	0.77	0.17	0.74	0.17	0.77	0.24	0.68	0.38
Instabilities	0.8	0.79	0.68	0.72	0.65	0.67	0.6	0.71
Average	0.66	0.4	0.68	0.46	0.7	0.48	0.64	0.54

**Table 3:** Lengths of the input vectors used for NPH depending on the anomaly type.

	Anomaly Type			
	Peaks	Bumps	Jumps	Instabilities
Average anomaly length	2.89	52.54	74.03	11.61
Input vector length for ANN-Center	9	158	222	35
Input vector length for ANN-Edge	6	105	148	23

**Table 4:** Lengths of the input vectors used for AEB and UNB depending on the anomaly type.

		Anomaly Type			
		Peaks	Bumps	Jumps	Instabilities
Average anomaly length		2.89	52.54	74.03	11.61
Input vector length		128	2048	4096	512

**Table 5:** Test results for the NPH method.

	Peak			Bump			Jump			Instabilities		
Error Type →	IoU	FPR	FNR	IoU	FPR	FNR	IoU	FPR	FNR	IoU	FPR	FNR
T	0.42	0.0012	0.41	0.75	0.0007	0.24	0.39	0.0016	0.6	0.78	0.0008	0.17
M	0.3	0.002	0.5	0.6	0.0131	0.22	0.0	0.0017	1.0	0.49	0.005	0.3
pH	0.44	0.0012	0.38	0.48	0.002	0.5	0.34	0.0042	0.64	0.72	0.0007	0.24
Average	0.39	0.0015	0.43	0.61	0.0053	0.32	0.24	0.0025	0.75	0.66	0.0022	0.24

**Table 6:** Test results for the AEB method.

	Peak			Bump			Jump			Instabilities		
Error Type →	IoU	FPR	FNR	IoU	FPR	FNR	IoU	FPR	FNR	IoU	FPR	FNR
T	0.9	0.0001	0.07	0.76	0.0052	0.17	0.43	0.0008	0.57	0.91	0.0006	0.04
M	0.71	0.0003	0.22	0.46	0.0321	0.21	0.02	0.0492	0.86	0.69	0.0021	0.19
pH	0.86	0.0001	0.09	0.48	0.0015	0.51	0.26	0.0008	0.74	0.85	0.0005	0.11
Average	0.82	0.0002	0.13	0.56	0.0129	0.3	0.23	0.0169	0.72	0.82	0.0011	0.12

**Table 7:** Test results for the UNB method.

	Peak			Bump			Jump			Instabilities		
Error Type →	IoU	FPR	FNR	IoU	FPR	FNR	IoU	FPR	FNR	IoU	FPR	FNR
T	0.89	0.0001	0.07	0.94	0.0006	0.05	0.48	0.0048	0.47	0.93	0.0003	0.04
M	0.67	0.0002	0.28	0.62	0.012	0.21	0.15	0.0104	0.63	0.72	0.0014	0.2
pH	0.85	0.0001	0.13	0.71	0.0011	0.28	0.51	0.0011	0.49	0.86	0.0004	0.11
Average	0.8	0.0001	0.16	0.76	0.0045	0.18	0.38	0.0054	0.53	0.84	0.0007	0.12

**Table 8:** Comparison of anomaly detection accuracy and processing time for the three considered methods.

Method →	NPH	AEB	UNB
Average IOU for test sensors	0.48	0.61	0.69
Number of multiplications per sample	632,800	22,716	5,209,632
Time complexity per sample (in sec.)	$4.1\cdot 10^{-6}$	$1.17\cdot 10^{-5}$	$1.17\cdot 10^{-5}$

**Table 9:** Comparison of AUC values between the AEB and UNB methods, calculated for anomaly detection ROC curves of test sensors.

	Peak			Bump			Jump			Instabilities		
Time Series →	T	M	pH	T	M	pH	T	M	pH	T	M	pH
AEB	1.0	0.96	0.99	0.95	0.92	0.89	0.75	0.72	0.74	0.99	0.97	0.98
UNB	0.98	0.92	0.96	0.95	0.88	0.87	0.82	0.86	0.77	0.99	0.94	0.97

## Data Availability

The original data presented in the study are openly available from IEEE DataPort at https://doi.org/10.21227/0j1h-ew11 (accessed on 21 August 2025).
